# Comparison of different tracers in sentinel lymph node detection for endometrial cancer: a systematic review and network meta-analysis

**DOI:** 10.1097/JS9.0000000000002064

**Published:** 2024-08-26

**Authors:** Chuanli Feng, Xuji Jiang, Lianlian Feng, Wanying Sun, Qingqing Liu, Yiping Hao, Baoxia Cui

**Affiliations:** Department of Obstetrics and Gynecology, Qilu Hospital of Shandong University, Shandong Province, People’s Republic of China

**Keywords:** endometrial cancer, network meta-analysis, sentinel lymph node, tracer

## Abstract

**Background:**

In the realm of endometrial cancer (EC) therapeutics and prognostic assessments, lymph nodes’ status is paramount. The sentinel lymph node (SLN) detection, recognized for its reliability, has been progressively adopted as a standard procedure, posing a compelling alternative to conventional systematic lymphadenectomy. However, there remains a lack of agreement on the most effective choice of tracers for this procedure.

**Objective:**

This investigation was dedicated to a comparative analysis of various tracers to identify the most effective combination that achieves the highest detection rate. This endeavor sought to enhance the efficacy of SLN biopsy in the surgical management of EC.

**Methods:**

A systematic review was conducted across multiple databases, including the Cochrane Central Register of Controlled Trials, PubMed, Web of Science, Embase, and clinicaltrials.gov, to analyze studies employing different tracers for SLN biopsy during surgery in EC. Using Bayesian network meta-analysis, the authors compared the total and bilateral detection rates of various tracers.

**Results:**

After screening 1431 articles, 11 studies, including 2699 participants, were selected in this network meta-analysis. The combination of radioactive isotopes and indocyanine green (ICG) emerged as the most efficacious method in total and bilateral detection rates, with the Surface Under the Cumulative Ranking Curve (SUCRA) scores of 80.00 and 86.36%, respectively. Additionally, carbon nanoparticles (CNPs) demonstrated superior performance in the detection of para-aortic lymph nodes with an SUCRA score of 97.77%.

**Conclusion:**

Network meta-analysis shows that the application of radioactive isotopes and ICG is the optimal tracer combination for SLN biopsy during surgery in EC.

## Introduction

Highlights

- The selection of tracers is the key to the detection of SLN in endometrial cancer, and the network meta-analysis was performed on the total and bilateral detection rates of various tracers.
- This article includes multiple tracer combinations, comparing novel tracers with traditional tracers such as CNPs and ICG.
- Compared to other tracers, the combination of 99mTc and ICG had a significant effect on the total and bilateral detection rates. The combination of 99mTc and ICG is a reliable tracer for the detection of SLN in endometrial cancer.

According to the International Federation of Gynecology and Obstetrics (FIGO) staging system, pathological information regarding the primary tumor and lymph node status guides the choice of adjuvant therapy and prognosis for patients with endometrial cancer (EC)^1^. Systematic lymphadenectomy, while increasing the incidence of intraoperative and postoperative complications, has not demonstrated a significant enhancement in survival rates, particularly in patients with low-risk EC^2^. The sentinel lymph node (SLN) biopsy is considered a more effective alternative to systematic lymphadenectomy, which accurately evaluates lymph nodes while minimizing collateral damage^3^. The SLN refers to the first station of lymphatic metastasis in primary malignant tumors, which can reflect tumor metastasis in distant lymphatic drainage areas. Tracers were injected into specific sites of the uterus so that the primary lymphatic pathways could be identified, to find more metastatic disease and reduce the additional risks of systematic lymphadenectomy. Therefore, accurate assessment of SLN status is crucial for formulating appropriate treatment strategies, and the choice of tracer may be the key to a successful SLN biopsy.

SLN biopsy mainly employs two distinct methods of visualization: dye-based staining and radioisotope tracking. Dye-based methods encompass a spectrum of colorants, including blue, black, and fluorescent dyes, which offer immediate visual cues during surgery. Indocyanine green (ICG) is the most widely used near-infrared fluorescent dye and is already considered a recommended tracer for cervical cancer^4^ and vulvar cancer^5^. It is distinguished by its ability to fluoresce under near-infrared excitation light, thereby offering high sensitivity, specificity, and detection rate. Blue dyes, including patent blue, isosulfan blue, and methylene blue, penetrate capillaries and fine lymphatic vessels, providing rapid visualization with quick transit to downstream lymph nodes. However, these dyes have the potential for allergic reactions and may induce lymphatic edema, which must be considered in clinical practice^6^. The black dye, specifically carbon nanoparticles (CNPs), has been successfully used as a new tracer for lymph node biopsy in EC, gastric cancer, colorectal cancer, breast cancer, and thyroid cancer^7–10^. With an average diameter of 150 nm, CNPs can blacken lymph nodes through lymphatic vessels with diameters of 120–500 nm, increasing the detection rate of small lymph nodes. It exhibits a unique capability to penetrate the interstitial spaces of the lymphatic capillary walls while being excluded from the blood capillaries. On the other hand, radioisotope tracing primarily utilizes technetium-99m (99mTC), which necessitates preoperative administration ~1 day before the surgical procedure. Detection is facilitated through nuclear imaging or gamma probe guidance. However, this approach introduces a layer of complexity in the operational process, contributing to increased therapeutic costs and heightened patient discomfort^11^. Recent advances have introduced combined or multiple tracer formulations to enhance detection rates, yet a consensus on the optimal staining method remains controversial.

In prior investigations, Smith *et al*.^12^ conducted a direct comparison between the detection rates of dye-based and radioisotopic tracers, as well as the combined use of both tracer categories. However, their study was primarily focused on evaluating the relative merits of ICG versus blue dye and did not extend to explore other specific combinations of tracers. Ilary Ruscito *et al*. analyzed tracers for uterine malignancies, they discovered that the detection rates of ICG and blue dye combined with 99mTc were similar. However, the above study primarily focused on patients with cervical cancer, limiting the generalizability of the conclusions to endometrial cancer, and the study included non-head-to-head experiments^13^. Consequently, the specificity of tracer types considered in prior studies was insufficient, with minimal exploration of tracers in head-to-head comparative studies and a lack of extensive direct and indirect comparisons of multiple combinations.

Therefore, this network meta-analysis, utilizing controlled trials, seeks to evaluate the effectiveness of different tracer combinations in SLN biopsy, analyzing variations in total and bilateral detection rates. The primary goal was to determine the optimal tracer combination that could enhance clinical outcomes.

## Material and methods

The systematic review and network meta-analysis were based on the Preferred Reporting Items for Systematic Reviews and Meta-Analyses (PRISMA, Supplemental Digital Content 1, http://links.lww.com/JS9/D360, Supplemental Digital Content 2, http://links.lww.com/JS9/D361) reporting guidelines^14^ and the methodological quality of systematic reviews (AMSTAR, Supplemental Digital Content 3, http://links.lww.com/JS9/D362)^15^. The registration of the study was completed on PROSPERO (CRD42023462651). As the research did not involve direct human subjects, institutional review board approval was not deemed necessary.

## Search strategy

We searched the Cochrane Register of Controlled Trials, PubMed, Web of Science, Embase, and Clinicaltrials.gov from the database inception to January 2024. The details of the search strategy are presented in the Supplemental search strategies (Supplemental Digital Content 4, http://links.lww.com/JS9/D363). To assess the diagnostic value of different tracers in SLN detection with EC, studies meeting the following criteria were included: (1) studies with a sample size of more than 10 patients with EC; (2) interventions involving any tracer or combination of tracers identified in the search; (3) prospective or retrospective literature; (4) outcomes including total detection rate, bilateral detection rate, or para-aortic detection rate; and (5) publications in English only.

Furthermore, we excluded studies that lacked original data, conference abstracts, case reports, literature reviews, guidelines, letters, and studies without full-text availability. We did a related article reading in a previous meta-analysis to ensure the comprehensive inclusion of studies.

Two authors independently screened the studies, and any discrepancies were resolved through consultation with a third author.

### Quality assessment

Non-randomized controlled trials (NRCTs) were independently evaluated using the validated ROBINS-I tool^16^ which includes seven domains: bias due to confounding, bias in the selection of participants, bias in classification of interventions, bias due to deviations from intended interventions, bias due to missing data, bias in the measurement of outcomes, and bias in the selection of the reported result. The risk of bias in each domain is rated as low, moderate, high, or critical. According to the ROBINS-I guidelines, a domain categorized as low bias risk requires a study equivalent to a well-conducted randomized trial in that domain. The QUADAS-2 tool may offer better accuracy for diagnostic studies, we believe that the ROBINS-I tool is more suitable for quality assessment in this research as the majority of studies retrieved were cohort studies that predominantly reported outcome detection rates rather than diagnostic accuracy (sensitivity and specificity). Retrospective cohort studies were considered observational intervention studies for diagnostic interventions.

Randomized controlled trials (RCTs) were evaluated using the Cochrane Risk of Bias 2 (RoB2) tool, including the following aspects: randomization process, deviations from intended interventions, missing data, outcome measurement, and selection of reporting results. They are set at three levels: low risk, some concerns, and high risk.

Two authors independently conducted the quality assessment of the literature, and any discrepancies were resolved through consultation with a third author.

### Data extraction

Before data collection, we created a data extraction form. For each included study, two authors independently extracted the following data: (1) author and publication details; (2) study design; (3) population; (4) type of tracers and sample size; (5) type of surgical approach; (6) patient characteristics (e.g. age and BMI); (7) tumor FIGO stage; (8) details of the injection technique; (9) SLN detection rate for each intervention; (10) bilateral detection rate; and (11) para-aortic detection rate. If multiple cancer types were reported simultaneously in this study, only data specific to endometrial cancer were extracted. Any discrepancies were resolved through consultation with a third author.

### Statistical analysis

The primary outcomes were the total detection rate and bilateral detection rate. The total detection rate was defined as the proportion of patients who had at least one SLN detected in the pelvis on either side, while the bilateral detection rate was defined as the proportion of patients with at least one SLN detected on each side of the pelvis. The secondary outcome was the para-aortic detection rate.

Stata17 is applied to analyze direct meta-analysis, make network evidence plots, and ‘comparison-adjusted’ funnel plots. Stata’s consistency model, which integrates measures of heterogeneity, inconsistency, and treatment effect size, enabled the conduct of a global consistency test. The significance of the model’s inconsistency was determined if the *P*-value fell below 0.05. For meta-analysis, funnel plots were created that included at least 10 original studies. Bayesian network meta-analysis was conducted using R software (including JAGS software) with BUGSnet and gemtc packages. The random effects Markov Chain Monte Carlo (MCMC) model was used. For binary outcomes, odds ratios (OR) or LogOR with their 95% CI were used as effect measures for the results. Trajectory and density plots were utilized to visually assess convergence. A potential scale reduction factor (PSRF) approaching or equal to 1 indicates good convergence of data. The Surface Under the Cumulative Ranking Curve (SUCRA) score was employed to rank the efficacy of each intervention and ascertain the optimal treatment. Interventions were scored for ranking according to their respective SUCRA scores, with higher scores indicating a higher position in the ranking.

## Results

### Literature search and Bias risk assessment

**Figure 1:**
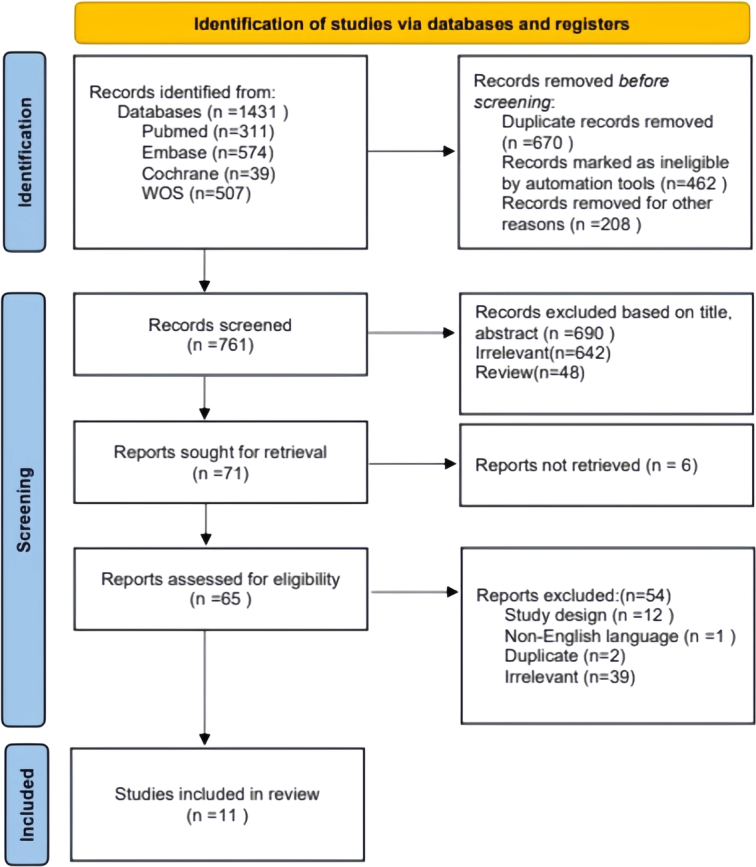
Preferred Reporting Items for Systematic Reviews and Meta-analyses (PRISMA) study flowchart.

**Figure 2:**
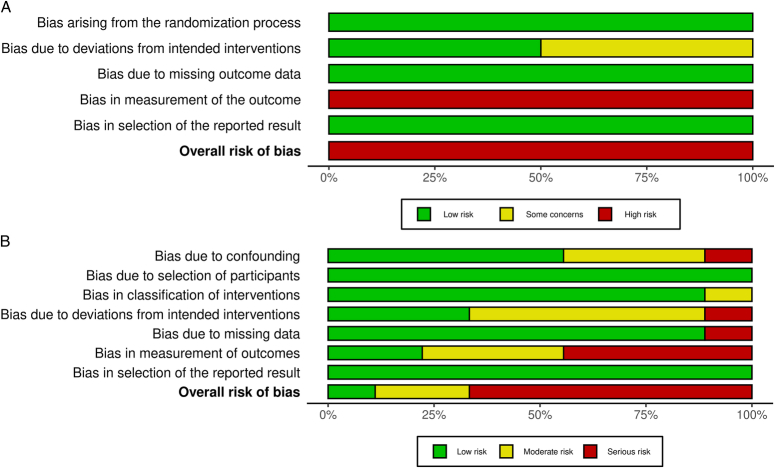
Summary for risk of bias of included clinical trials. (A) RCTs were evaluated using RoB2 tools; (B) NRCTs were evaluated using ROBINS-I tools. The green symbols represent low risk of bias, the yellow symbols represent unclear risk of bias, and the red symbols represent high risk of bias.

A total of 1431 articles were screened, resulting in 11 articles for inclusion in the study based on predetermined criteria. The systematic literature search and study selection process is depicted in Figure 1. In the assessment of bias risk, NRCTs were evaluated, with six studies deemed to have a ‘severe’ risk of bias, two studies with a ‘moderate’ risk, and one study with a ‘low’ risk. RCTs were both considered to be at ‘high’ risk of bias, the details of the quality assessment are shown in Figure 2 and Supplementary Fig. 1 (Supplemental Digital Content 4, http://links.lww.com/JS9/D363). In general, there was a high risk of bias in the included studies, mainly due to the predominance of NRCTs (82%) and differences in observation methods of tracer imaging, which potentially compromised the blinding of surgeons to the intervention.

### Study characteristics

The included studies are summarized in Supplementary Table 1 (Supplemental Digital Content 4, http://links.lww.com/JS9/D363). A total of 11 studies involving 2699 participants were included, comprising seven prospective and four retrospective articles. There were seven different tracer combinations, namely ICG, 99mTc+ICG, CNPs, blue dye, 99mTc+blue, blue + ICG + 99mTc, and 99mTc. Among them, three articles compared blue dye and ICG^17–19^, three articles compared 99mTc+ICG vs ICG^20–22^, two articles each compared 99mTC+blue vs ICG^19,23^ and 99mTc+blue vs 99mTc+ICG^24,25^. The remaining CNPs vs ICG^26^, 99mTC vs ICG^22^, and blue+ICG+99mTc vs 99mTc+ICG^27^ have only one article each. During surgery in EC, 1106 patients (41%) underwent ICG imaging. Surgical approaches encompassed laparoscopy (1643 patients), robotic methods (530 patients), and undergoing open surgery (214 patients), except for two articles that did not specify the distribution of surgical methods.

### Pair-wise meta-analysis

Pairwise meta-analysis and forest maps were used to compare two or more studies and forest maps were presented in Supplementary Fig. 2 (Supplemental Digital Content 4, http://links.lww.com/JS9/D363).

#### Total detection rate

The results of the conventional pair-wise meta-analysis indicate that, in the comparison of the total detection rate across 11 articles and 7 interventions, there is a significant increase in the total detection rate of ICG over blue dye during SLN biopsy (*n*=3, OR=0.23, 95% CI=[0.10–0.52], *I* ^2^ =0%). However, for the remaining interventions, no significant difference is observed between the two methods. Overall, there is low statistical heterogeneity, except for the comparison of 99mTc+ICG vs ICG (*I* ^2^=86%).

#### Bilateral detection rate

The results show that ICG is more efficacious than 99mTC (*n*=1, OR=0.57, 95% CI=[0.43–0.74]), blue dye (*n*=3, OR=0.31, 95% CI=[0.17–0.59], *I* ^2^=35.3%) and 99mTC+blue (*n*=2, OR=0.41, 95% CI=[0.20–0.81], *I* ^2^ =41.3%) for SLN biopsy. For 99mTC+blue vs 99mTC+ICG (*n*=2, OR=0.31, 95% CI=[0.16–0.63]), the analysis data shows a significant increase for 99mTC+ICG during SLN biopsy. Comparisons of other groups were not statistically significant. In general, except for 99mTc+ICG vs ICG (*I* ^2^ = 75%), the statistical heterogeneity is small.

#### Para-aortic detection rate

The results of the traditional pair-wise meta-analysis suggest that compared with 99mTC (*n*=1, OR=0.15, 95% CI= [0.10–0.24]), ICG is more efficacious and there is no statistical difference between the other groups.

### Network meta-analysis

The results from the inconsistent model show a *P*-value ≥0.05, prompting the utilization of a consistency model for Bayesian network meta-analysis. With a PSRF value of 1.01, indicating good convergence, the consistency model demonstrates favorable statistical properties. Detailed visualization of the convergence plot and density plot are shown in Supplemental Figure 3 (Supplemental Digital Content 4, http://links.lww.com/JS9/D363).

#### Total detection rate

**Figure 3:**
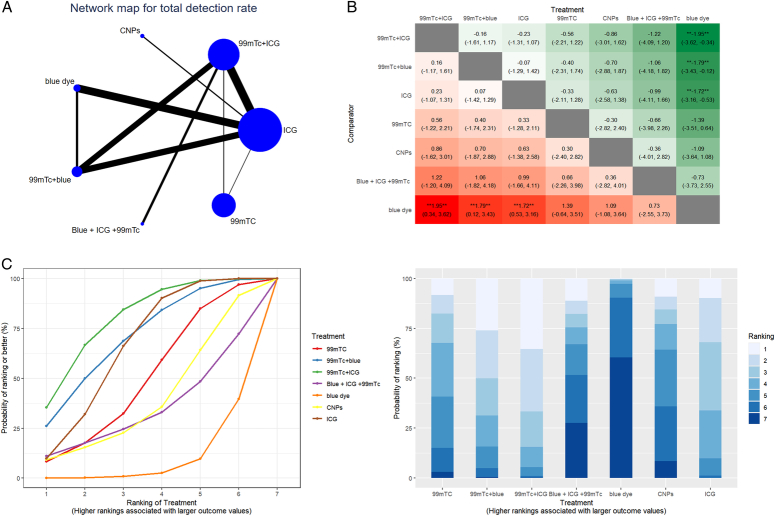
The results of network meta-analysis of total detection rate. (A) Geometry of comparisons in the network meta-analysis of total detection rate. (B) Comparison of total detection rate of different tracer combinations in network meta-analysis. (C) Ranking of different tracers with endometrial cancer assessed using surface under the cumulative ranking (SUCRA) values.

On total detection rate, ICG demonstrates a strong correlation with blue dye but shows minimal correlation with CNPs or 99mTC. Compared with blue dye, 99mTC+ICG (LogOR = 1.95, 95% CI=[0.34–3.62]), 99mTC+blue (LogOR = 1.79, 95% CI=[0.12–3.43]) and ICG (LogOR=1.72, 95% CI=[0.53–3.16]) exhibit significant impacts on the total detection rate. Network meta-analysis involves seven different tracer combinations, 99mTc+ICG has the highest SUCRA score, followed by 99mTc+blue and ICG. Detailed results of the total detection rate are shown in Figure 3 and Supplemental Table 2 (Supplemental Digital Content 4, http://links.lww.com/JS9/D363).

#### Bilateral detection rate

**Figure 4:**
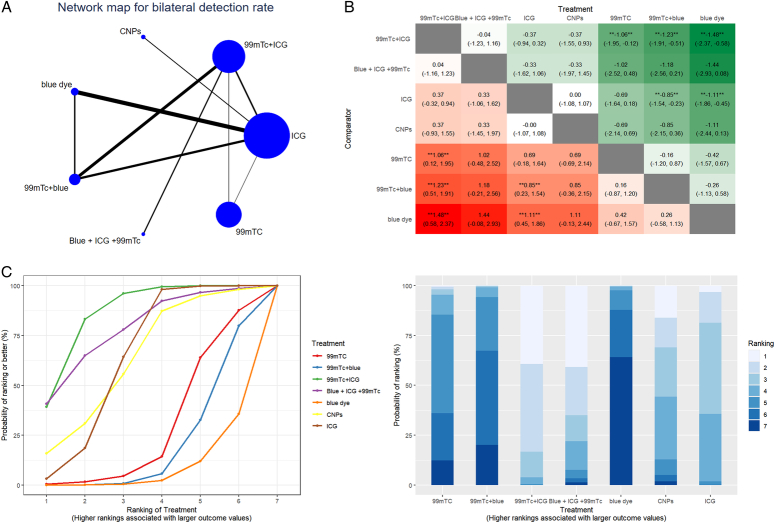
The results of network meta-analysis of bilateral detection rate. (A) Geometry of comparisons in the network meta-analysis of bilateral detection rate. (B) Comparison of bilateral detection rate of different tracer combinations in network meta-analysis. (C) Ranking of different tracers with endometrial cancer assessed using surface under the cumulative ranking (SUCRA) values.

On bilateral detection rate, ICG has a close relationship with blue dye, while it has little relationship with CNPs or 99mTC. Compared with 99mTC, 99mTC+ICG (LogOR=1.06, 95% CI=[0.12–1.95]) has a significant effect on the bilateral detection rate. Compared with 99mTC+blue, both 99mTC+ICG (LogOR = 1.23, 95% CI=[0.51–1.91]) and ICG (LogOR=0.85, 95% CI=[0.23–1.54]) have significant effects. 99mTC+ICG (LogOR = 1.48, 95% CI=[0.58–2.37]) and ICG (LogOR =1.11, 95% CI=[0.45–1.86]) have better effects than blue dye. The ranking probability shows that 99mTc+ICG has the highest SUCRA score, and blue dye is the lowest. Detailed results of the bilateral detection rate are shown in Figure 4 and Supplemental Table 2 (Supplemental Digital Content 4, http://links.lww.com/JS9/D363).

Regarding the bilateral detection rate, CNPs exhibited the highest sucra value. Detailed results can be found in Supplemental Figure 4 (Supplemental Digital Content 4, http://links.lww.com/JS9/D363) and Appendix 1 (Supplemental Digital Content 4, http://links.lww.com/JS9/D363).

### Publication bias

**Figure 5:**
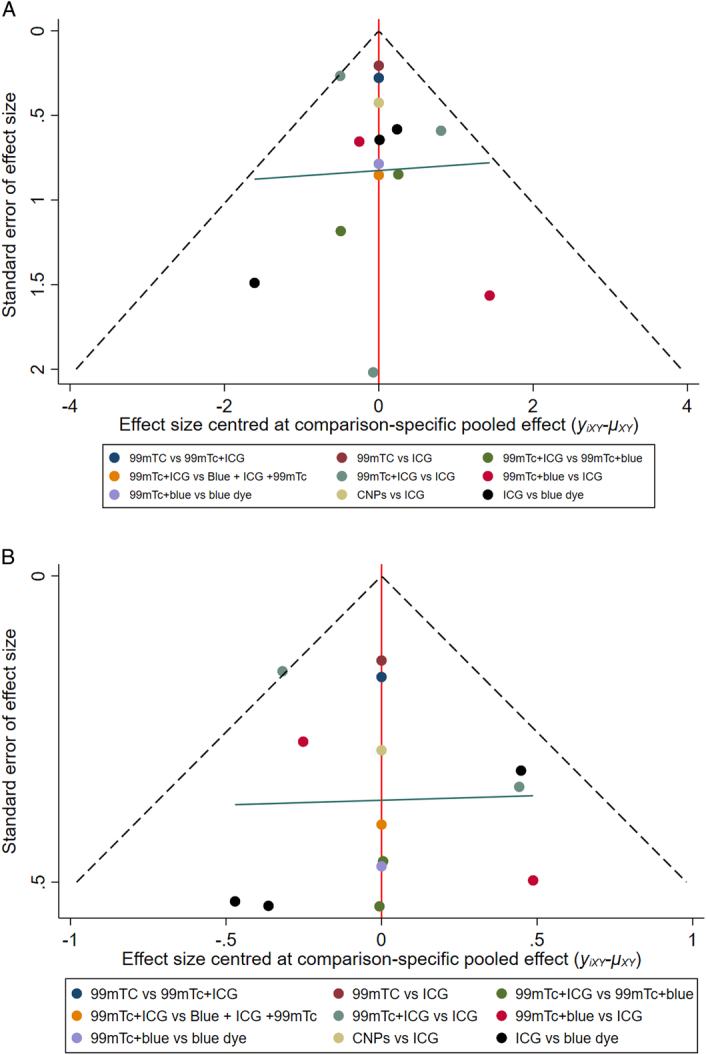
Comparison-adjusted funnel plots for comparison of multiple tracers in patients with endometrial cancer. (A) total detection rate; (B) bilateral detection rate.

‘Comparison-adjusted’ funnel plots are shown in Figure 5. Overall, in the funnel plots adjusted for comparison, no significant asymmetry was found in the total and bilateral detection rates, indicating no evidence of publication bias.

## Discussion

In this study, we performed a network meta-analysis to evaluate the effectiveness of various tracer combinations in the SLN biopsy for EC. A thorough literature review was performed, and data from 11 studies, including 7 tracer combinations, were synthesized. Through comparisons of the total detection rate, bilateral detection rate, and para-aortic detection rate across multiple tracer combinations, we aimed to identify an optimal tracer combination that could enhance the detection rate of SLN in EC, and facilitate the early identification of metastatic disease. Our results indicate that all interventions in this network meta-analysis have positive impacts on SLN detection, with the 99mTc and ICG combination showing particular efficacy.

In recent years, with the development of minimally invasive surgery, ICG has been reported in some areas, such as hepatic blood flow assessment, choroidal blood flow assessment, and cardiac output measurement^28,29^. Served as a fluorescent dye, ICG is widely used in clinical practice due to its low cost, visualization of lymphatic vessels with real-time visual guidance, and few side effects^30^. In traditional meta-analysis, ICG demonstrates a higher total detection rate and bilateral detection rate compared to other tracers. Despite this, it is important to note that ICG has certain limitations^31^. An important aspect is that when excising tissue is imaged by ICG, the final pathology determines only adipose tissue and not lymph nodes. Michael Frumovitz *et al*.^32^ reported an incidence rate of 5–6% and the status of the entire half of the pelvis cannot be accurately assessed. Another drawback is the rapid diffusion of ICG and its potential to swiftly traverse the SLN to reach secondary and tertiary lymph nodes, potentially leading to inadvertent excision of nonsentinel lymph nodes by surgeons. Furthermore, the limited ability of ICG to penetrate tissue results in a low detection rate in obese patients (BMI >34)^33^. Unlike radiotracers, ICG is not sequestered by macrophages within the lymph nodes, which necessitates its intraoperative injection to capitalize on its rapid lymphatic drainage. This characteristic precludes the acquisition of preoperative lymphatic mapping, which is instrumental in surgical planning.

99mTC can be used for SLN biopsy before surgery through SPECT/CT or planar lymphatic scintillation imaging, which can help surgeons predict the patient’s surgical status and SLN number^34^. For some reason, the surgeons need a long time to dissect the lymph node, 99mTc can fix the ICG to avoid staining the remaining lymph nodes^35^. It is their complementary characteristics that make their combination clinically practical and provide the basis for our research findings. In network meta-analysis, the rank probability analysis indicates that this combination is most likely to be ranked first, with a SUCRA score of 80.00% for the total detection rate and 86.36% for the bilateral detection rate, thereby suggesting that the combination of 99mTc and ICG represents the optimal tracer combination for preoperative SLN detection. These findings are corroborated in the work by Anna Torrent *et al*.^35^, which supports the notion that the combination of 99mTc and ICG is a superior tracer. However, Cabrera *et al*.^36^ found in a retrospective discussion that the combination of 99mTc and ICG during surgery in EC does not improve the detection rate of early EC. Therefore, further prospective studies should be conducted to definitively rule out this association.

In the analysis of para-aortic detection rate, CNPs stand out as the most significant tracer, with the highest SUCRA score of 97.77%, significantly outperforming the second-ranked tracer (64.27% ) (Supplemental Fig. 4, Supplemental Digital Content 4, http://links.lww.com/JS9/D363). It is important to acknowledge that not all studies included in the analysis reported a para-aortic detection rate. Limited available data was collected suggesting that latent para-aortic metastatic lymph nodes may be present. This situation may have affected the accuracy of our meta-analysis. However, in early-stage EC, para-aortic lymph node metastasis is rare in the absence of pelvic lymph node disease (1%). If the para-aortic SLN fails, localization of the SLN in the pelvis is sufficient for most patients, as isolated para-aortic metastases are uncommon^37^. Although CNPs have a high detection rate in para-aortic detection, the inclusion of literature on CNPs in this study was limited, and supporting data was restricted. Therefore, further research is needed to investigate the potential of this promising tracer to improve the lymph node detection rate.

To our knowledge, this is the inaugural study to synthesize existing evidence on the efficiency of different tracers for SLN biopsy in patients with EC. Different from traditional pair-wise meta-analysis, this network meta-analysis incorporates both direct and indirect evidence. Moreover, this network meta-analysis exclusively encompasses RCTs and NRCTs, including both prospective and retrospective studies. To ensure methodological rigor and high-quality evidence, we have excluded studies where the same cohort received multiple interventions.

However, this network meta-analysis had several limitations. First, although we included a network meta-analysis of studies with different FIGO stages, stages Ⅲ–Ⅳ were in the minority, so it is prudent to generalize the results of this network meta-analysis to all patients with EC. Second, the injection site has always been a major problem in SLN biopsy. The uterus is a central organ with a complex lymphatic drainage system^38^. Common injection sites include the cervix, base, pelvic floor, etc.^35,39^. Our study found inconsistencies between the injection site and injection method, which may impact detection efficiency. However, the included studies did not provide effective data on different injection sites; the effect of injection sites on SLN biopsy efficiency was not discussed in this study. Additionally, in the actual practice of SLN biopsy, professional knowledge and technical details of the surgeons are crucial. Finally, although we have carefully searched for various tracers, the language was limited to English, potentially missing relevant studies published in other languages. Moreover, gray literature was not searched.

## Conclusion

The network meta-analysis revealed that the combination of 99mTc and ICG holds a distinct advantage for the visualization of SLN in EC. The tissue penetration capability of ICG, coupled with the stabilizing effect of 99mTc on ICG, indeed enhances the accuracy of SLN biopsy. Well-designed RCTs with definitive intervention protocols could further substantiate the efficacy of various tracers in SLN biopsy, offering more reliable guidance for clinical practice.

## Ethical approval

None. This study has no ethical implications.

## Consent

None.

## Source of funding

None.

## Author contribution

C.F.: data analysis or interpretation and writing the paper; X.J.: study concept or design and data collection; L.F.: writing the paper; W.S.: data collection; Q.L. and Y.H.: quality assessment; B.C.: review the article.

## Conflicts of interest disclosure

The authors declare no conflicts of interest.

## Research registration unique identifying number (UIN)

Registration of the study was completed on PROSPERO (CRD42023462651).

## Guarantor

Baoxia Cui.

## Data availability statement

All data generated and analyzed during this study are included in these published articles.

## Provenance and peer review

Not commissioned, externally peer-reviewed.

## Supplementary Material
