# Supplementary material for: Comparison of different tracers in sentinel lymph node detection for endometrial cancer: a systematic review and network meta-analysis
Source: Int J Surg. 2024 Aug 26;111(1):1397–406. doi: 10.1097/JS9.0000000000002064 (PMC11745743; doi:10.1097/JS9.0000000000002064)
Supplement: Supplementary file 2 [file js9-111-1397-s002.docx]

**Identification of studies via databases and registers**

Records removed *before screening*:

Duplicate records removed (n =670 )

Records marked as ineligible by automation tools (n=462 )

Records removed for other reasons (n =208 )

Records identified from:

Databases (n =1431 )

Pubmed (n=311)

Embase (n=574)

Cochrane (n=39)

WOS (n=507)

**Identification**

Records screened

(n =761)

Records excluded based on title,

abstract (n =690 ) Irrelevant(n=642)

Review(n=48)

Reports sought for retrieval

(n =71)

Reports not retrieved (n = 6)

**Screening**

Reports assessed for eligibility

(n =65 )

Reports excluded:(n=54)

Study design (n =12 )

Non-English language (n =1 )

Duplicate (n=2)

Irrelevant (n=39)

Studies included in review

(n =11 )

**Included**

*Consider, if feasible to do so, reporting the number of records identified from each database or register searched (rather than the total number across all databases/registers).

**If automation tools were used, indicate how many records were excluded by a human and how many were excluded by automation tools.

*From:*  Page MJ, McKenzie JE, Bossuyt PM, Boutron I, Hoffmann TC, Mulrow CD, et al. The PRISMA 2020 statement: an updated guideline for reporting systematic reviews. BMJ 2021;372:n71. doi: 10.1136/bmj.n71

For more information, visit: <http://www.prisma-statement.org/>
