# Supplementary material for: Comparison of different tracers in sentinel lymph node detection for endometrial cancer: a systematic review and network meta-analysis
Source: Int J Surg. 2024 Aug 26;111(1):1397–406. doi: 10.1097/JS9.0000000000002064 (PMC11745743; doi:10.1097/JS9.0000000000002064)
Supplement: Supplementary file 4 [file js9-111-1397-s004.docx]

Supplemental Online Content

Supplemental search strategies

Supplementary Fig1. Summary for risk of bias of included clinical trials.

Supplementary Fig2.Forest plots from pairwise comparisons of treatment agents.

Supplemental Fig3.Convergence diagram and density diagram of Network-meta analysis.

Supplemental Fig4.The results of network meta-analysis of para-aortic detection rate.

Supplemental Appendix1.The results of secondary outcome para-aortic detection rate in network-meta analysis.

Supplementary Table 1.Characteristics of the included trials.

Supplemental Table 2. Ranking of different tracers with endometrial cancer assessed using surface under the cumulative ranking (SUCRA) values.

**Supplemental search strategies**

PubMed

"endometrial neoplasms"[MeSH Terms] OR ("endometrial"[All Fields] AND "neoplasms"[All Fields]) OR "endometrial neoplasms"[All Fields] OR ("endometrial"[All Fields] AND "cancer"[All Fields]) OR "endometrial cancer"[All Fields] OR ("endometrial neoplasms"[MeSH Terms] OR ("endometrial"[All Fields] AND "neoplasms"[All Fields]) OR "endometrial neoplasms"[All Fields] OR ("endometrial"[All Fields] AND "neoplasm"[All Fields]) OR "endometrial neoplasm"[All Fields]) OR ("endometrial neoplasms"[MeSH Terms] OR ("endometrial"[All Fields] AND "neoplasms"[All Fields]) OR "endometrial neoplasms"[All Fields] OR ("neoplasm"[All Fields] AND "endometrial"[All Fields]) OR "neoplasm endometrial"[All Fields]) OR ("endometrial neoplasms"[MeSH Terms] OR ("endometrial"[All Fields] AND "neoplasms"[All Fields]) OR "endometrial neoplasms"[All Fields] OR ("neoplasms"[All Fields] AND "endometrial"[All Fields]) OR "neoplasms endometrial"[All Fields]) OR ("endometrial neoplasms"[MeSH Terms] OR ("endometrial"[All Fields] AND "neoplasms"[All Fields]) OR "endometrial neoplasms"[All Fields] OR ("endometrial"[All Fields] AND "carcinoma"[All Fields]) OR "endometrial carcinoma"[All Fields]) OR ("endometrial neoplasms"[MeSH Terms] OR ("endometrial"[All Fields] AND "neoplasms"[All Fields]) OR "endometrial neoplasms"[All Fields] OR ("carcinoma"[All Fields] AND "endometrial"[All Fields]) OR "carcinoma endometrial"[All Fields]) OR ("endometrial neoplasms"[MeSH Terms] OR ("endometrial"[All Fields] AND "neoplasms"[All Fields]) OR "endometrial neoplasms"[All Fields] OR ("carcinomas"[All Fields] AND "endometrial"[All Fields]) OR "carcinomas endometrial"[All Fields]) OR ("endometrial neoplasms"[MeSH Terms] OR ("endometrial"[All Fields] AND "neoplasms"[All Fields]) OR "endometrial neoplasms"[All Fields] OR ("endometrial"[All Fields] AND "carcinomas"[All Fields]) OR "endometrial carcinomas"[All Fields]) OR ("endometrial neoplasms"[MeSH Terms] OR ("endometrial"[All Fields] AND "neoplasms"[All Fields]) OR "endometrial neoplasms"[All Fields] OR ("endometrial"[All Fields] AND "cancer"[All Fields]) OR "endometrial cancer"[All Fields]) OR ("endometrial neoplasms"[MeSH Terms] OR ("endometrial"[All Fields] AND "neoplasms"[All Fields]) OR "endometrial neoplasms"[All Fields] OR ("cancer"[All Fields] AND "endometrial"[All Fields]) OR "cancer endometrial"[All Fields]) OR ("endometrial neoplasms"[MeSH Terms] OR ("endometrial"[All Fields] AND "neoplasms"[All Fields]) OR "endometrial neoplasms"[All Fields] OR ("cancers"[All Fields] AND "endometrial"[All Fields]) OR "cancers endometrial"[All Fields]) OR ("endometrial neoplasms"[MeSH Terms] OR ("endometrial"[All Fields] AND "neoplasms"[All Fields]) OR "endometrial neoplasms"[All Fields] OR ("endometrial"[All Fields] AND "cancers"[All Fields]) OR "endometrial cancers"[All Fields]) OR ("endometrial neoplasms"[MeSH Terms] OR ("endometrial"[All Fields] AND "neoplasms"[All Fields]) OR "endometrial neoplasms"[All Fields] OR ("endometrium"[All Fields] AND "cancer"[All Fields]) OR "endometrium cancer"[All Fields]) OR ("endometrial neoplasms"[MeSH Terms] OR ("endometrial"[All Fields] AND "neoplasms"[All Fields]) OR "endometrial neoplasms"[All Fields] OR ("cancer"[All Fields] AND "endometrium"[All Fields]) OR "cancer endometrium"[All Fields]) OR ("endometrial neoplasms"[MeSH Terms] OR ("endometrial"[All Fields] AND "neoplasms"[All Fields]) OR "endometrial neoplasms"[All Fields] OR ("cancers"[All Fields] AND "endometrium"[All Fields]) OR "cancers endometrium"[All Fields]) OR ("endometrial neoplasms"[MeSH Terms] OR ("endometrial"[All Fields] AND "neoplasms"[All Fields]) OR "endometrial neoplasms"[All Fields] OR ("cancer"[All Fields] AND "endometrium"[All Fields]) OR "cancer of the endometrium"[All Fields]) OR ("endometrial neoplasms"[MeSH Terms] OR ("endometrial"[All Fields] AND "neoplasms"[All Fields]) OR "endometrial neoplasms"[All Fields] OR ("carcinoma"[All Fields] AND "endometrium"[All Fields]) OR "carcinoma of endometrium"[All Fields]) OR ("endometrial neoplasms"[MeSH Terms] OR ("endometrial"[All Fields] AND "neoplasms"[All Fields]) OR "endometrial neoplasms"[All Fields] OR ("endometrium"[All Fields] AND "carcinoma"[All Fields]) OR "endometrium carcinoma"[All Fields]) OR ("endometrial neoplasms"[MeSH Terms] OR ("endometrial"[All Fields] AND "neoplasms"[All Fields]) OR "endometrial neoplasms"[All Fields] OR ("endometrium"[All Fields] AND "carcinomas"[All Fields]) OR "endometrium carcinomas"[All Fields]) OR ("endometrial neoplasms"[MeSH Terms] OR ("endometrial"[All Fields] AND "neoplasms"[All Fields]) OR "endometrial neoplasms"[All Fields] OR ("cancer"[All Fields] AND "endometrium"[All Fields]) OR "cancer of endometrium"[All Fields]) OR ("endometrial neoplasms"[MeSH Terms] OR ("endometrial"[All Fields] AND "neoplasms"[All Fields]) OR "endometrial neoplasms"[All Fields] OR ("endometrium"[All Fields] AND "cancers"[All Fields]) OR "endometrium cancers"[All Fields])

AND

"sentinel lymph node"[MeSH Terms] OR ("sentinel"[All Fields] AND "lymph"[All Fields] AND "Node"[All Fields]) OR "sentinel lymph node"[All Fields] OR ("sentinel lymph node"[MeSH Terms] OR ("sentinel"[All Fields] AND "lymph"[All Fields] AND "Node"[All Fields]) OR "sentinel lymph node"[All Fields] OR ("lymph"[All Fields] AND "Node"[All Fields] AND "sentinel"[All Fields]) OR "lymph node sentinel"[All Fields]) OR ("sentinel lymph node"[MeSH Terms] OR ("sentinel"[All Fields] AND "lymph"[All Fields] AND "Node"[All Fields]) OR "sentinel lymph node"[All Fields] OR ("lymph"[All Fields] AND "nodes"[All Fields] AND "sentinel"[All Fields]) OR "lymph nodes sentinel"[All Fields]) OR ("sentinel lymph node"[MeSH Terms] OR ("sentinel"[All Fields] AND "lymph"[All Fields] AND "Node"[All Fields]) OR "sentinel lymph node"[All Fields] OR ("sentinel"[All Fields] AND "lymph"[All Fields] AND "nodes"[All Fields]) OR "sentinel lymph nodes"[All Fields]) OR ("sentinel lymph node"[MeSH Terms] OR ("sentinel"[All Fields] AND "lymph"[All Fields] AND "Node"[All Fields]) OR "sentinel lymph node"[All Fields] OR ("sentinel"[All Fields] AND "Node"[All Fields]) OR "sentinel node"[All Fields]) OR ("Node"[All Fields] AND ("sentinel"[All Fields] OR "sentinels"[All Fields])) OR (("node s"[All Fields] OR "nodes"[All Fields]) AND ("sentinel"[All Fields] OR "sentinels"[All Fields])) OR (("sentinel"[All Fields] OR "sentinels"[All Fields]) AND ("node s"[All Fields] OR "nodes"[All Fields])) OR ("sentinel lymph node biopsy"[MeSH Terms] OR ("sentinel"[All Fields] AND "lymph"[All Fields] AND "Node"[All Fields] AND "biopsy"[All Fields]) OR "sentinel lymph node biopsy"[All Fields]) OR ("sentinel lymph node biopsy"[MeSH Terms] OR ("sentinel"[All Fields] AND "lymph"[All Fields] AND "Node"[All Fields] AND "biopsy"[All Fields]) OR "sentinel lymph node biopsy"[All Fields] OR ("lymph"[All Fields] AND "Node"[All Fields] AND "biopsy"[All Fields] AND "sentinel"[All Fields]) OR "lymph node biopsy sentinel"[All Fields]) OR ("sentinel lymph node biopsy"[MeSH Terms] OR ("sentinel"[All Fields] AND "lymph"[All Fields] AND "Node"[All Fields] AND "biopsy"[All Fields]) OR "sentinel lymph node biopsy"[All Fields] OR ("biopsy"[All Fields] AND "sentinel"[All Fields] AND "lymph"[All Fields] AND "Node"[All Fields]) OR "biopsy sentinel lymph node"[All Fields])

AND

(("carbon"[MeSH Terms] OR "carbon"[All Fields] OR "carbons"[All Fields] OR "carbon s"[All Fields] OR "carbonates"[MeSH Terms] OR "carbonates"[All Fields] OR "carbonate"[All Fields] OR "carbonated"[All Fields] OR "carbonating"[All Fields] OR "carbonation"[All Fields] OR "carboneous"[All Fields] OR "carbonization"[All Fields] OR "carbonizations"[All Fields] OR "carbonize"[All Fields] OR "carbonized"[All Fields] OR "carbonizing"[All Fields] OR "carbonous"[All Fields] OR "fizzy"[All Fields]) AND ("nanoparticle s"[All Fields] OR "nanoparticles"[MeSH Terms] OR "nanoparticles"[All Fields] OR "nanoparticle"[All Fields])) OR "CNPs"[All Fields] OR "indocyanine green"[MeSH Terms] OR ("indocyanine"[All Fields] AND "green"[All Fields]) OR "indocyanine green"[All Fields] OR "ICG"[All Fields] OR ("indocyanine green"[MeSH Terms] OR ("indocyanine"[All Fields] AND "green"[All Fields]) OR "indocyanine green"[All Fields] OR ("green"[All Fields] AND "indocyanine"[All Fields]) OR "green indocyanine"[All Fields]) OR ("indocyanine green"[MeSH Terms] OR ("indocyanine"[All Fields] AND "green"[All Fields]) OR "indocyanine green"[All Fields] OR "wofaverdin"[All Fields]) OR ("indocyanine green"[MeSH Terms] OR ("indocyanine"[All Fields] AND "green"[All Fields]) OR "indocyanine green"[All Fields] OR "vophaverdin"[All Fields]) OR "ujoviridin"[All Fields] OR ("indocyanine green"[MeSH Terms] OR ("indocyanine"[All Fields] AND "green"[All Fields]) OR "indocyanine green"[All Fields] OR "vofaverdin"[All Fields]) OR ("indocyanine green"[MeSH Terms] OR ("indocyanine"[All Fields] AND "green"[All Fields]) OR "indocyanine green"[All Fields] OR ("cardio"[All Fields] AND "green"[All Fields]) OR "cardio green"[All Fields]) OR ("indocyanine green"[MeSH Terms] OR ("indocyanine"[All Fields] AND "green"[All Fields]) OR "indocyanine green"[All Fields] OR ("cardio"[All Fields] AND "green"[All Fields]) OR "cardio green"[All Fields]) OR ("indocyanine green"[MeSH Terms] OR ("indocyanine"[All Fields] AND "green"[All Fields]) OR "indocyanine green"[All Fields] OR "cardiogreen"[All Fields]) OR "methylene blue"[MeSH Terms] OR ("methylene"[All Fields] AND "blue"[All Fields]) OR "methylene blue"[All Fields] OR ("methylene blue"[MeSH Terms] OR ("methylene"[All Fields] AND "blue"[All Fields]) OR "methylene blue"[All Fields] OR ("blue"[All Fields] AND "methylene"[All Fields]) OR "blue methylene"[All Fields]) OR ("methylene blue"[MeSH Terms] OR ("methylene"[All Fields] AND "blue"[All Fields]) OR "methylene blue"[All Fields] OR ("methylthioninium"[All Fields] AND "chloride"[All Fields]) OR "methylthioninium chloride"[All Fields]) OR ("methylene blue"[MeSH Terms] OR ("methylene"[All Fields] AND "blue"[All Fields]) OR "methylene blue"[All Fields] OR ("methylthionine"[All Fields] AND "chloride"[All Fields]) OR "methylthionine chloride"[All Fields]) OR ("methylene blue"[MeSH Terms] OR ("methylene"[All Fields] AND "blue"[All Fields]) OR "methylene blue"[All Fields] OR ("swiss"[All Fields] AND "blue"[All Fields]) OR "swiss blue"[All Fields]) OR ("methylene blue"[MeSH Terms] OR ("methylene"[All Fields] AND "blue"[All Fields]) OR "methylene blue"[All Fields] OR ("blue"[All Fields] AND "swiss"[All Fields])) OR ("methylene blue"[MeSH Terms] OR ("methylene"[All Fields] AND "blue"[All Fields]) OR "methylene blue"[All Fields] OR "basic blue 9"[All Fields]) OR ("methylene blue"[MeSH Terms] OR ("methylene"[All Fields] AND "blue"[All Fields]) OR "methylene blue"[All Fields] OR "blue 9 basic"[All Fields]) OR ("methylene blue"[MeSH Terms] OR ("methylene"[All Fields] AND "blue"[All Fields]) OR "methylene blue"[All Fields] OR "methylene blue n"[All Fields]) OR ("methylene blue"[MeSH Terms] OR ("methylene"[All Fields] AND "blue"[All Fields]) OR "methylene blue"[All Fields]) OR ("methylene blue"[MeSH Terms] OR ("methylene"[All Fields] AND "blue"[All Fields]) OR "methylene blue"[All Fields] OR "chromosmon"[All Fields]) OR ("methylene blue"[MeSH Terms] OR ("methylene"[All Fields] AND "blue"[All Fields]) OR "methylene blue"[All Fields] OR ("urolene"[All Fields] AND "blue"[All Fields]) OR "urolene blue"[All Fields]) OR ("methylene blue"[MeSH Terms] OR ("methylene"[All Fields] AND "blue"[All Fields]) OR "methylene blue"[All Fields] OR ("blue"[All Fields] AND "urolene"[All Fields])) OR "technetium"[MeSH Terms] OR "technetium"[All Fields] OR ("technetium"[MeSH Terms] OR "technetium"[All Fields] OR ("technetium"[All Fields] AND "99m"[All Fields]) OR "technetium 99m"[All Fields]) OR ("technetium"[MeSH Terms] OR "technetium"[All Fields] OR ("99m"[All Fields] AND "technetium"[All Fields]) OR "99m technetium"[All Fields]) OR ("isosulfane"[All Fields] AND "blue"[All Fields]) OR ("ieee int conf systems biol"[Journal] OR "isb"[All Fields]) OR ("iso sulfan blue"[Supplementary Concept] OR "iso sulfan blue"[All Fields] OR "isosulfan blue"[All Fields])

Cochrane

#1MeSH descriptor: [Endometrial Neoplasms] explode all trees

#2Endometrial Neoplasm OR Neoplasm, Endometrial OR Neoplasms, Endometrial OR Endometrial Carcinoma OR Carcinoma, Endometrial OR Carcinomas, Endometrial OR Endometrial Carcinomas OR Endometrial Cancer OR Cancer, Endometrial OR Cancers, Endometrial OR Endometrial Cancers OR Endometrium Cancer OR Cancer, Endometrium OR Cancers, Endometrium OR Cancer of the Endometrium OR Carcinoma of Endometrium OR Endometrium Carcinoma OR Endometrium Carcinomas OR Cancer of Endometrium OR Endometrium Cancers

#3 Sentinel Lymph Node OR Lymph Node, Sentinel OR Lymph Nodes, Sentinel OR Sentinel Lymph Nodes OR Sentinal Node OR Node, Sentinal OR Nodes, Sentinal OR Sentinal Nodes OR Sentinel Lymph Node Biopsy OR Lymph Node Biopsy, Sentinel OR Biopsy, Sentinel Lymph Node

#4 MeSH descriptor: [Sentinel Lymph Node] explode all trees

#5 #1 OR #2

#6 #3 OR #4

#7 carbon nanoparticles OR CNPs

#8 indocyanine green OR Green, Indocyanine OR Wofaverdin OR Vophaverdin OR Ujoveridin OR Vofaverdin OR Cardio-Green OR Cardio Green OR Cardiogreen

#9 methylene blue OR Blue, Methylene OR Methylthioninium Chloride OR Methylthionine Chloride OR Swiss Blue OR Blue, Swiss OR Basic Blue 9 OR Blue 9, Basic OR Methylene Blue N OR Blue N, Methylene OR Chromosmon OR Urolene Blue OR Blue, Urolene

#10 Technetium OR Technetium 99m OR 99m, Technetium

#11 isosulfane blue OR isosulfan blue

#12 #11 OR #7 OR #8 OR #9 OR #10

#13 #5 AND #12 # 6

Embase

'endometrium tumor'/exp OR 'endometrial neoplasm':ab,ti OR 'endometrial carcinoma':ab,ti OR 'endometrial carcinomas':ab,ti OR 'endometrial cancer':ab,ti OR 'endometrial cancers':ab,ti OR 'endometrium cancer':ab,ti OR 'cancer of the endometrium':ab,ti OR 'carcinoma of endometrium':ab,ti OR 'endometrium carcinoma':ab,ti OR 'endometrium carcinomas':ab,ti OR 'cancer of endometrium':ab,ti OR 'endometrium cancers':ab,ti OR 'endometrial neoplasms'

AND

'sentinel lymph node'/exp OR 'sentinel lymph node':ab,ti OR 'lymph node, sentinel':ab,ti OR 'lymph nodes, sentinel':ab,ti OR 'sentinel lymph nodes':ab,ti OR 'sentinal node':ab,ti OR 'node, sentinal':ab,ti OR 'nodes, sentinal':ab,ti OR 'sentinal nodes':ab,ti OR 'sentinel lymph node biopsy':ab,ti OR 'lymph node biopsy, sentinel':ab,ti OR 'biopsy, sentinel lymph node':ab,ti

AND

'carbon nanoparticle'/exp OR 'carbon nanoparticle' OR 'carbon nanoparticles':ab,ti OR 'cnps':ab,ti OR 'cnp':ab,ti OR 'indocyanine green'/exp OR 'Green, Indocyanine':ab,ti:ab,ti OR Wofaverdin:ab,ti OR Vophaverdin:ab,ti OR Ujoveridin:ab,ti OR Vofaverdin:ab,ti OR Cardio-Green:ab,ti OR 'Cardio Green':ab,ti OR Cardiogreen:ab,ti OR 'methylene blue'/exp OR 'Blue, Methylene':ab,ti OR 'Methylthioninium Chloride':ab,ti OR 'Methylthionine Chloride':ab,ti OR 'Swiss Blue':ab,ti OR 'Blue, Swiss':ab,ti OR 'Basic Blue 9':ab,ti OR 'Blue 9, Basic':ab,ti OR 'Methylene Blue N':ab,ti OR 'Blue N, Methylene':ab,ti OR Chromosmon:ab,ti OR 'Urolene Blue':ab,ti OR 'Blue, Urolene':ab,ti OR 'technetium'/exp OR 'Technetium 99m':ab,ti OR '99m, Technetium':ab,ti OR 'isosulfane blue':ab,ti OR 'isosulfan blue':ab,ti

Web of science

TS=(Endometrial Neoplasms OR Endometrial Neoplasm OR Endometrial Carcinoma OR Endometrial Carcinomas OR Endometrial Cancer OR Endometrial Cancers OR Endometrium Cancer OR Cancer of the Endometrium OR Carcinoma of Endometrium OR Endometrium Carcinoma OR Endometrium Carcinomas OR Cancer of Endometrium OR Endometrium Cancers)

AND

TS=(Sentinel Lymph Node OR Sentinel Lymph Nodes OR Sentinal Nodes OR Sentinel Lymph Node Biopsy)

AND

TS=(carbon nanoparticles) OR TS=(indocyanine green OR Wofaverdin OR Vophaverdin OR Ujoveridin OR Vofaverdin OR Cardio-Green OR Cardio Green OR Cardiogreen) OR TS=(methylene blue OR Methylthioninium Chloride OR Methylthionine Chloride OR Swiss Blue OR Basic Blue 9 OR Methylene Blue N OR Chromosmon) OR TS=(Technetium OR Technetium 99m) OR TS=(isosulfane blue OR isosulfan blue)

**Supplementary Fig1. Summary for risk of bias of included clinical trials.** The green symbols represent low risk of bias, the yellow symbols represent unclear risk of bias, and the red symbols represent high risk of bias. (a) RCT were evaluated using RoB2 tools. (b) NRCT were evaluated using ROBINS-I tools.

(a)


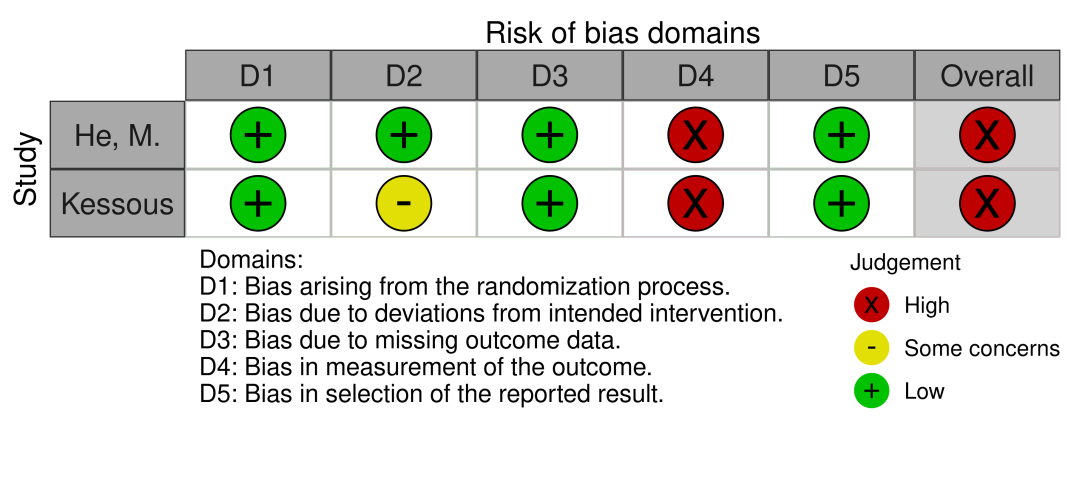


(b)


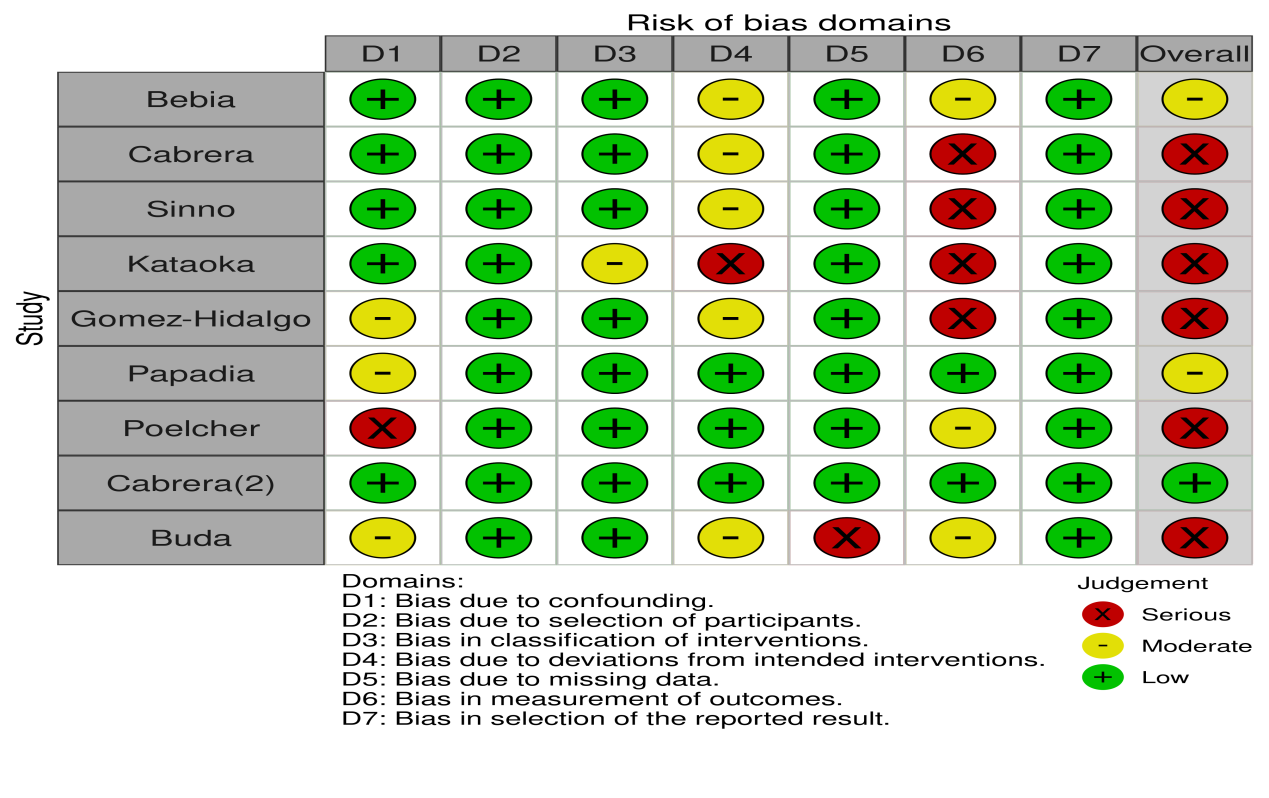


**Supplementary Fig2.Forest plots from pairwise comparisons of treatment agents.** The result of a single study was identified as direct evidence when pairwise meta-analysis for a specific comparison was not attainable. (A) total detection rate, (B) bilateral detection rate, (C) para-aortic detection rate.

1. total detection rate


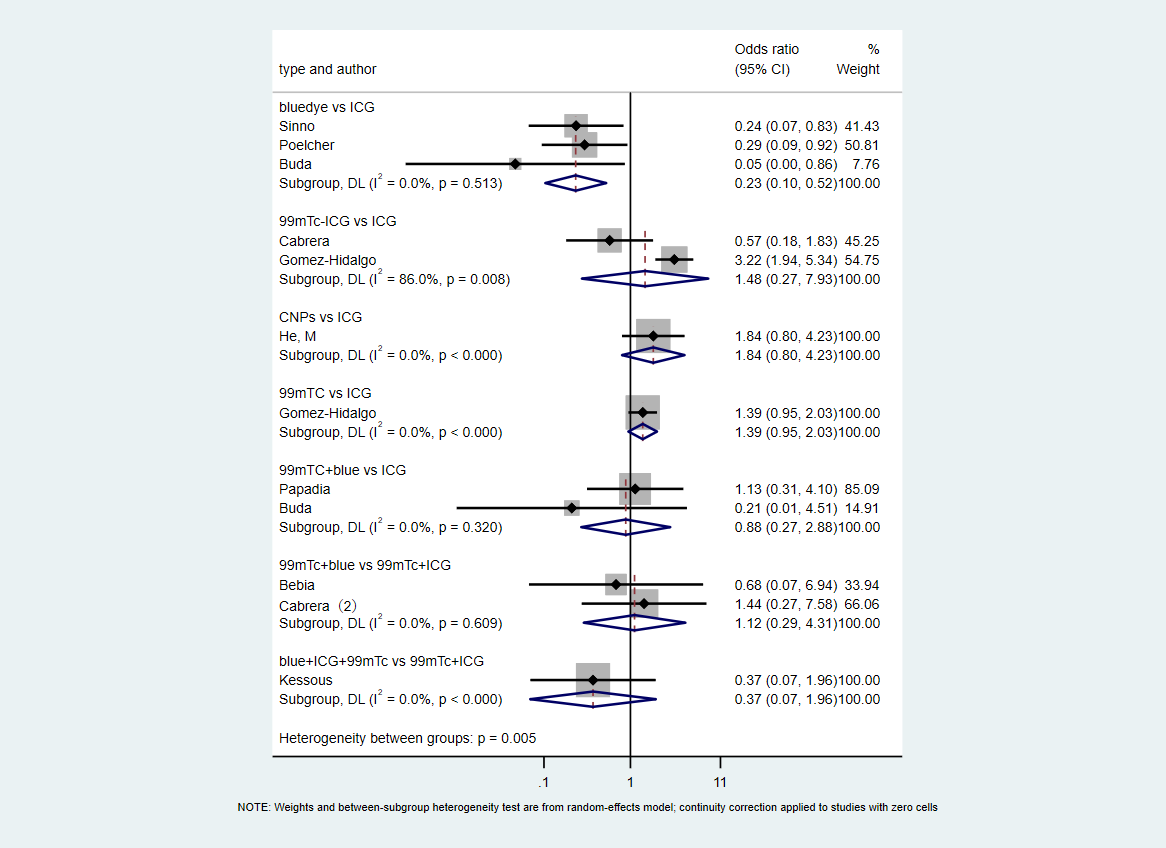


1. bilateral detection rate


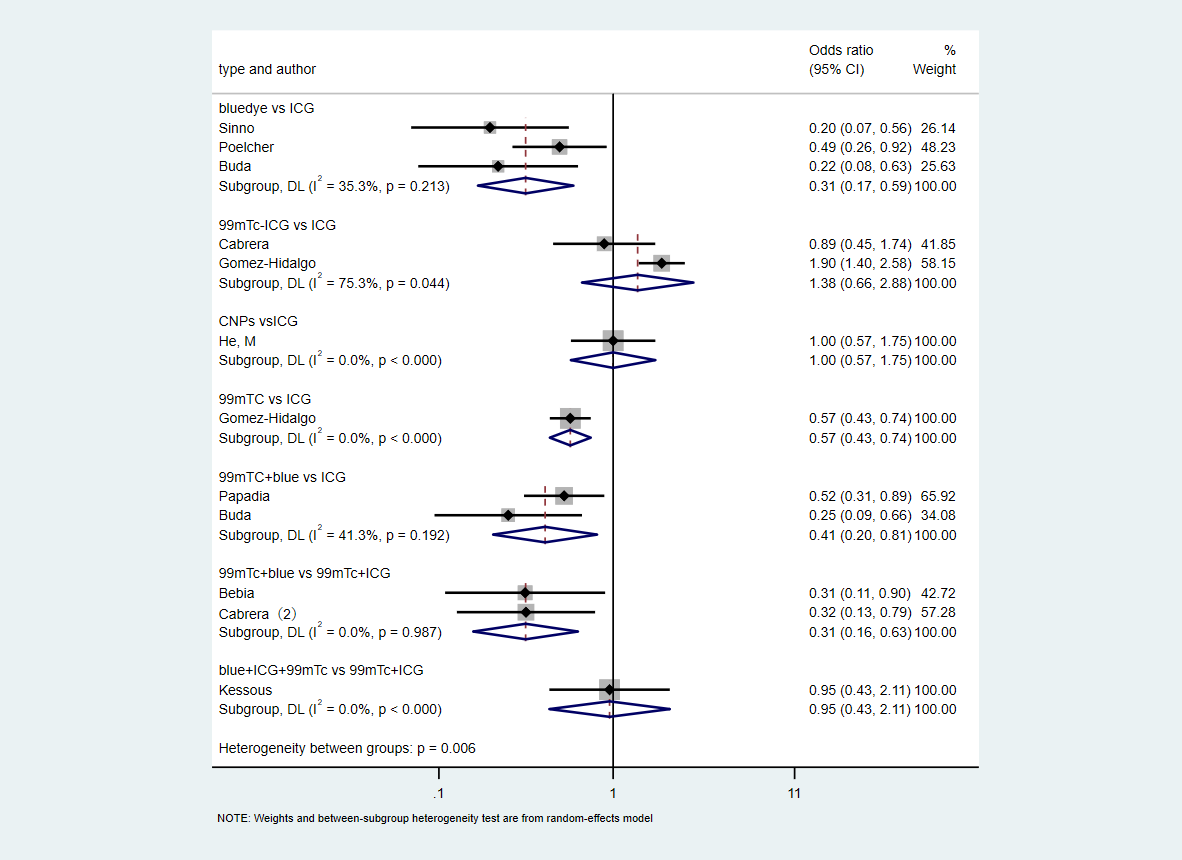


(C) para-aortic detection rate

The results of the traditional pairwise meta-analysis suggest that 99mTC vs ICG (n=1,OR=0.15, 95%CI= [0.10,0.24])，the analysis data showed a significant increase for ICG SLN mapping，There was no statistical difference between the other groups.The heterogeneity of 99mTC+ICG reached 89.9%, and the remaining heterogeneity was small.Due to the limited number of articles,and this outcome para-aortic detection rate indicator is not available in every article, so the number of articles is relatively small, with only one study except 99mTC+ICG vs ICG.


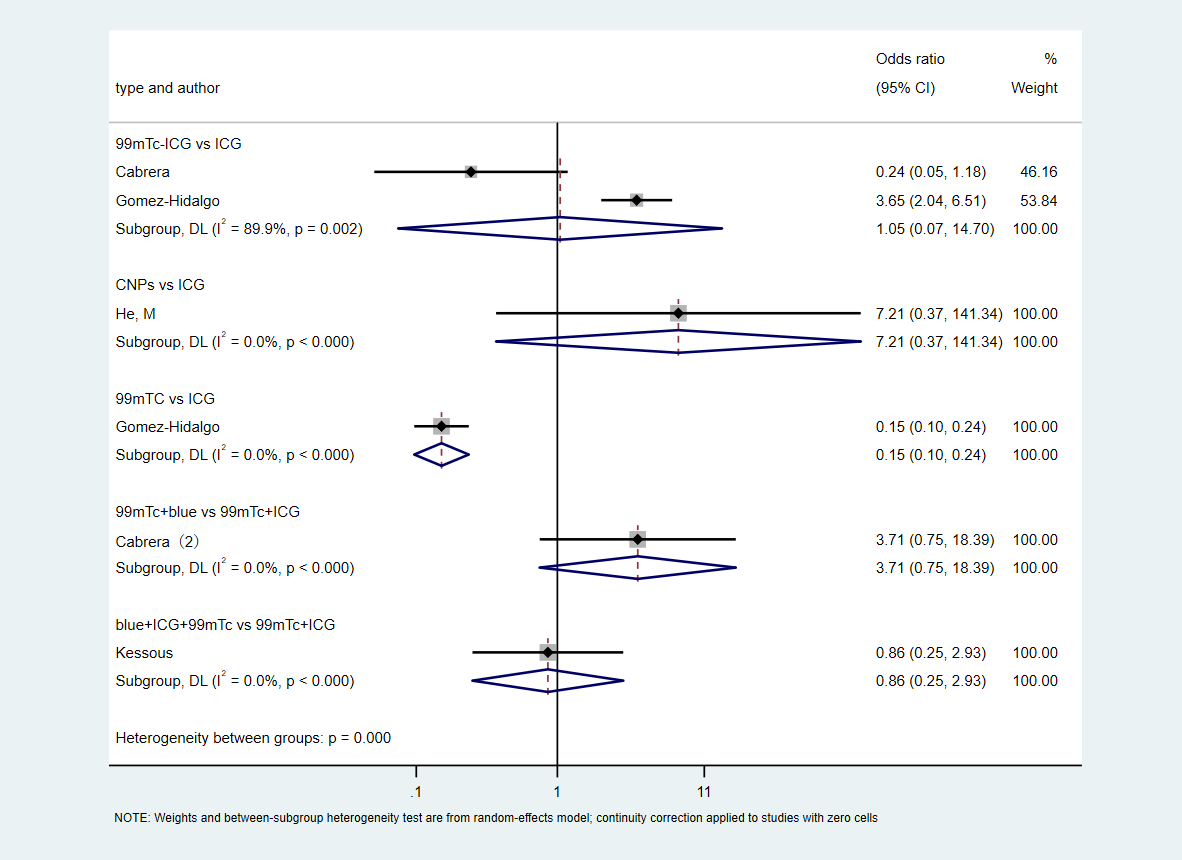


**Supplemental Figure 3.Convergence diagram and density diagram of Network-meta analysis.**


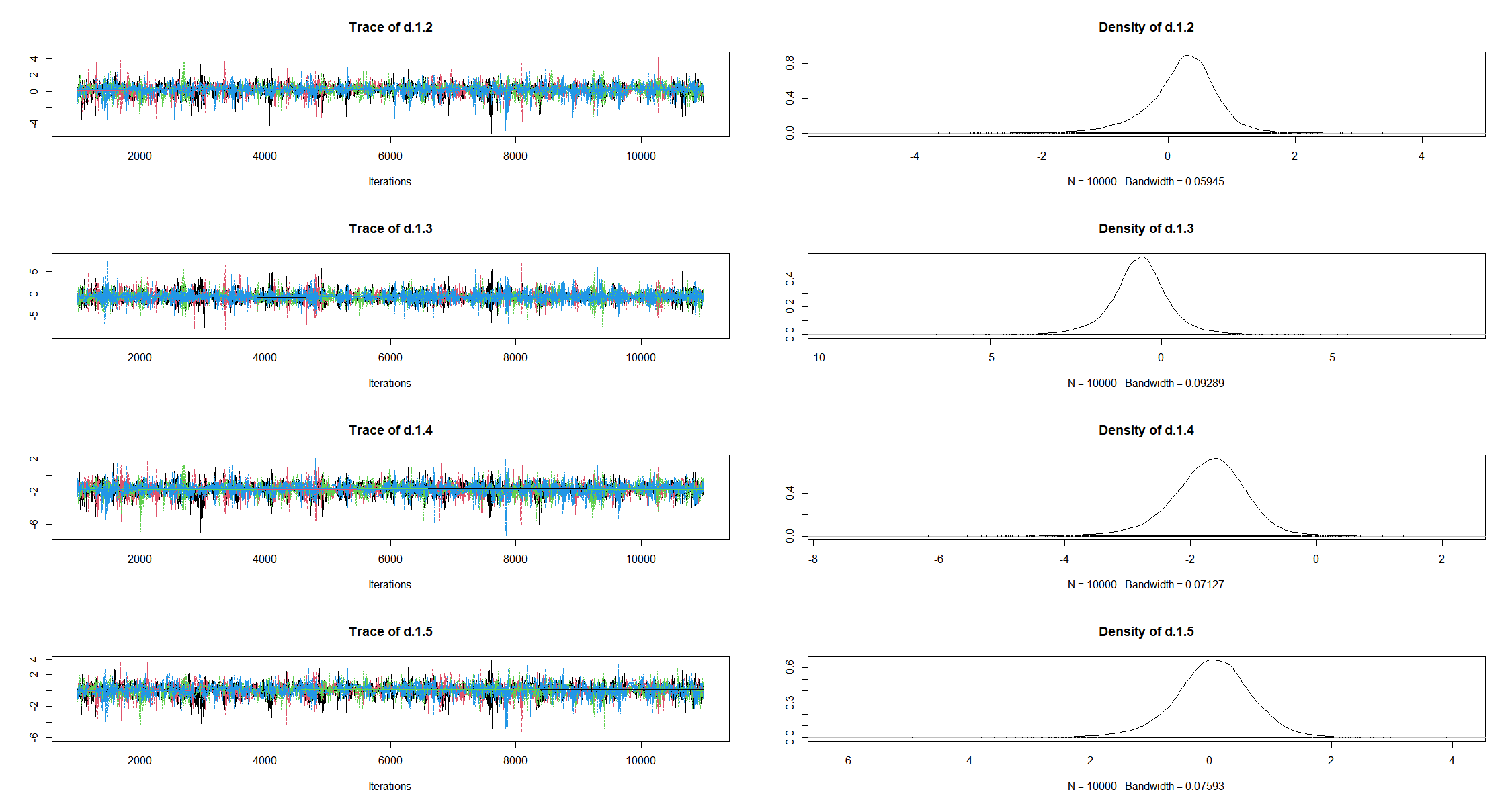

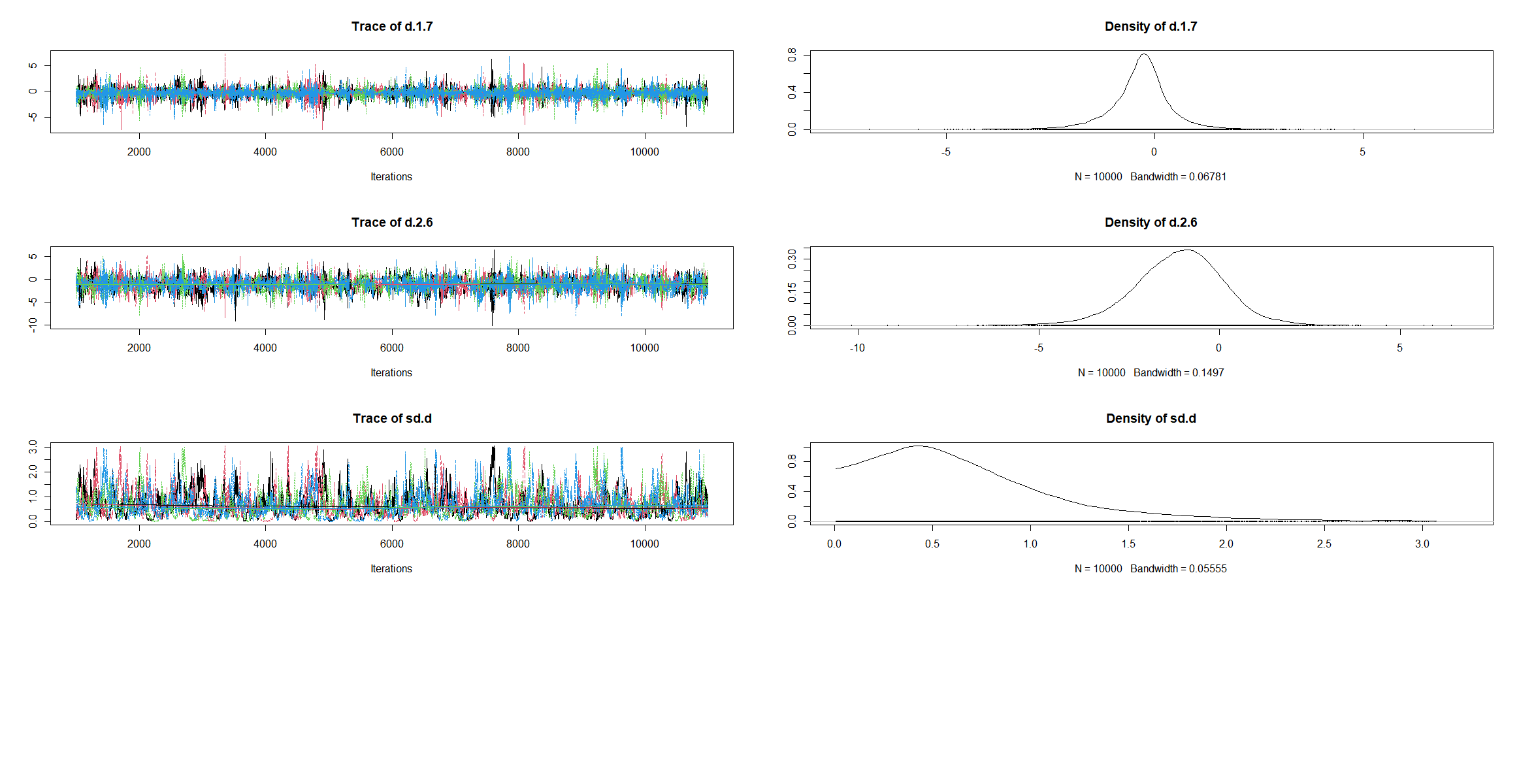

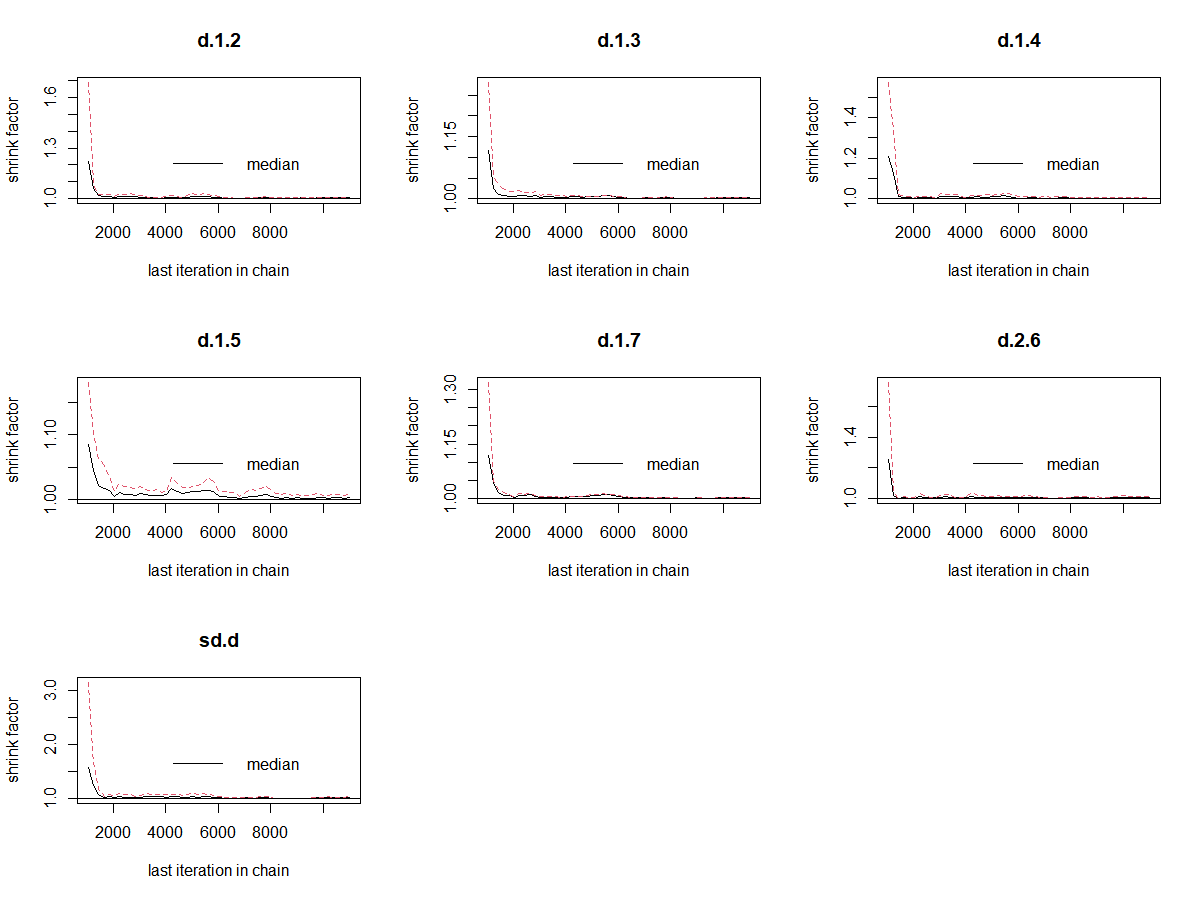


**Supplemental Fig4.The results of network meta-analysis of para-aortic detection rate.** (a) Geometry of comparisons in the network meta-analysis of para-aortic detection rate. (b) Cumulative ranking of tracers for patients with endometrial cancer based on the probability of their effects on outcomes of para-aortic detection rate. (c) Ranking of different tracers with endometrial cancer assessed using surface under the cumulative ranking (SUCRA) values. (d) Comparison of para-aortic detection rate of different tracer combinations in network meta-analysis.

1. (b)

| Treatment | Para-aortic detection rates  (%) |
| --- | --- |
| ICG | 32.78 |
| 99mTc+ICG | 35.65 |
| carbon nanoparticles（CNPs） | 97.77 |
| blue dye | - |
| 99mTc+blue | 64.27 |
| blue + ICG +99mTc | 32.45 |
| 99mTC | 37.08 |


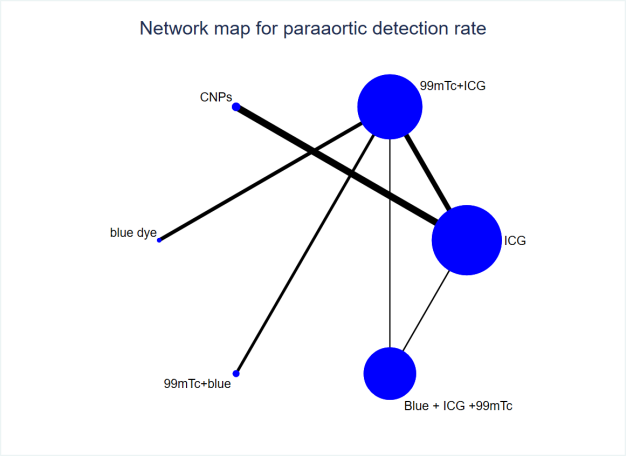


(c)


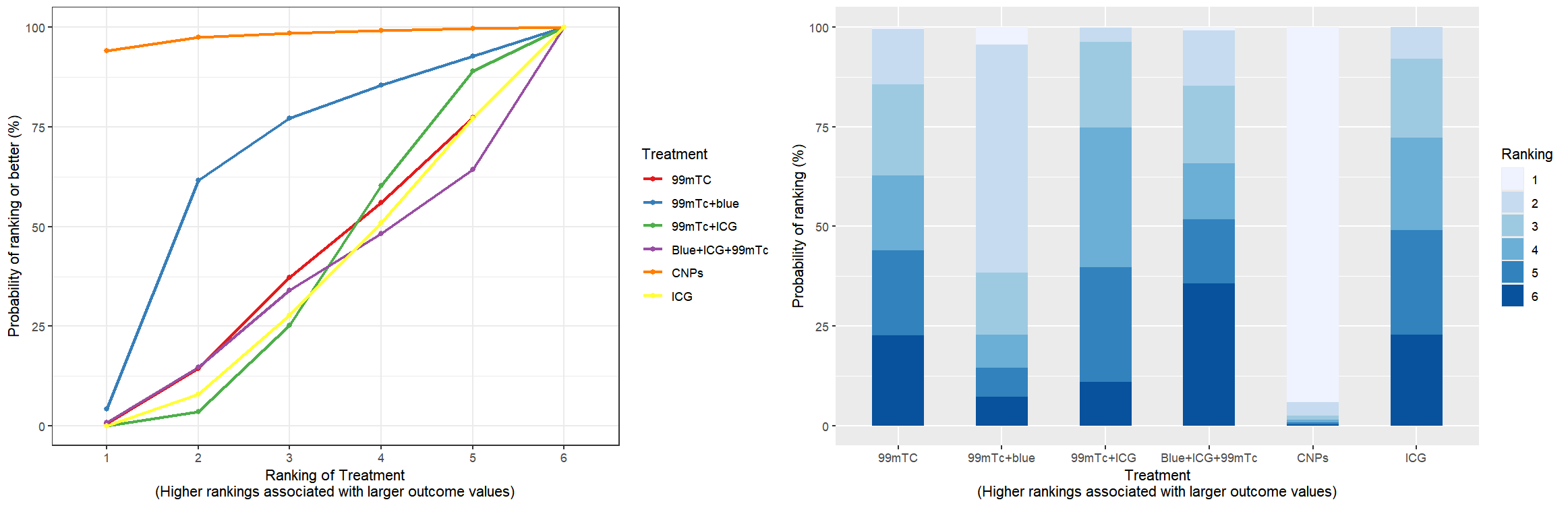


(d)


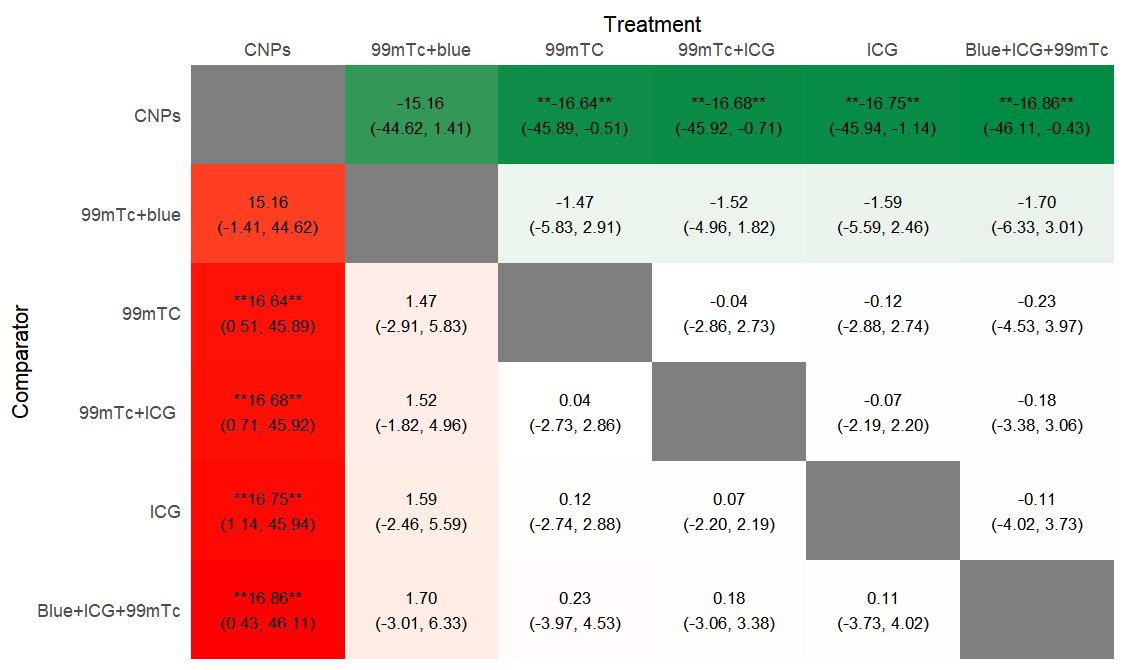


**Supplemental Appendix1.The results of secondary outcome para-aortic detection rate in network-meta analysis.**

A network diagram of the para-aortic detection rate is shown in Supplemental Fig4(a) , which shows a close relationship between ICG and CNP , a distant relationship with Blue + ICG+ 99mTc. Supplemental Fig4(c) provides ranking probabilities for different tracer interventions. The results showed that CNPs had the largest sucra value. The relative effects of various tracers on para-aortic detection rate(Supplemental Fig4d). A total of 6 interventions were compared. CNPs makes sense compared to any other group except 99mTC+blue(LogOR = 11.53, 95%CI=[-1.73,29.71]).

**Supplementary Table 1.Characteristics of the included trials.**

ICG:indocyanine green; ISB:a kind of blue dye,isosulfan blue; 99mTc:technetium-99m radiocolloid.

|  | Author | Inclusion period | Study design | Number | Age | BMI | FIGO stage | Treatment1 | Treatment2 | Treatment3 | Surgical procedure | Details of injection method |
| --- | --- | --- | --- | --- | --- | --- | --- | --- | --- | --- | --- | --- |
| 1 | Sinno,2014 | 2012.9-2014.3 | prospective | 71 | 62.25 | 33.55 | ⅠA-ⅣB | ICG | ISB | NA | Robotic-assisted  laparoscopy71 | cervix at the 3 and 9 o'clock |
| 2 | Poelcher,2021 | 2017.3-2020.1 | retrospective | 198 | 65.74 | NA | ⅠA-ⅣB | blue dye | ICG | NA | Most with Laparoscopy | intracervically at 3 and 9 o'clock both superficially and in 1 cm depth |
| 3 | Buda,2016 | 2010.10-2015.5 | retrospective | 114 | NA | NA | 1A2–1B1 | 99mTc+blue | blue dye | ICG | Laparoscopy  Laparotomy | cervix 3- and 9o’clock positions,a depth of 1 cm into the stroma, and the submucosal layer on the right and the left of the cervix |
| 4 | Cabrera,2021 | 2015.2-2020.6 | retrospective | 180 | 62.43 | 29.47 | ⅠA-ⅣB | ICG | 99mTc+ICG | NA | Laparoscopy103(57.2%)  Robotic-assisted76(42.2%)  Open1(0.6%) | intracervically with 4 injections of 0.2 mL at 3 and 9 o'clock, deep (1–1.5 cm) and superficially (1–2mm) |
| 5 | Kataoka,2016 | 2009.4-2012.12 | prospective | 55 | 53.33 | NA | ⅠA-ⅢC2 | ICG | 99mTc+ICG | NA | Open surgery55 | sub-endometrium at 5 points |
| 6 | Gomez-  Hidalgo,2022 | 2015.1-2022.1 | retrospective | 1221 | 63 | 29.3 | Ⅰ-Ⅱ | ICG | 99mTc+ICG | 99mTC | Laparoscopy985(80.7%)  Robotic-assisted187(15.3%)  Laparotomy49(4.0%) | cervical\uterine\both |
| 7 | Papadia,2017 | 2010-2016 | retrospective | 342 | 63.07 | 31.24 | ⅠA-Ⅳ | 99mTc + blue | ICG | NA | Laparoscopy313(91.52%)  Open29(8.48%) | cervix at the 3 and 9 o'clock |
| 8 | Bebia,2019 | 2017.2-2019.3 | prospective | 71 | 62.4 | NA | NA | 99mTc+  blue | 99mTc+ICG | NA | Laparotomy3(4.2%)，  Minimally invasive68 (95.8%) | NA |
| 9 | Cabrera（2）,2020 | 2017.2-2019.7 | prospective | 84 | 63.1 | 30.7 | ⅠA-ⅢC2 | 99mTc+blue | 99mTc+ICG | NA | Laparotomy5(3.6%)  Laparoscopy40(47.6%)  Robotic39(48.8%) | intracervically at 3 and 9 o’clock, deep (1–1.5 cm) and superficially (1–2 mm), |
| 10 | He, M,2023 | 2020.8-2022.4 | prospective | 206 | 56.36 | 25.94 | ⅠA-Ⅲ | ICG | CNPs | NA | Laparoscopy134(65.04%),  Laparotomy72(34.96%) | Cervix at 3 and 9 o'clock both deeply (1 cm) and superficially (1–3 mm) |
| 11 | Kessous,2019 | 2015.6-2017.5 | prospective | 157 | 64.79 | 34 | ⅠA-Ⅳ | 99mTc+ICG | Blue+ICG +99mTc | NA | Robotic surgical157 | cervix at the 3 and 9 o'clock,superficially into the cervical submucosa and deep into the stroma of the cervix |

**Supplemental Table 2. Ranking of different tracers with endometrial cancer assessed using surface under the cumulative ranking (SUCRA) values**

| Treatment | Total detection rate  (%) | Bilateral detection rate  (%) |
| --- | --- | --- |
| ICG | **66.18** | **64.04** |
| 99mTc+ICG | **80.00** | **86.36** |
| carbon nanoparticles（CNPs） | 39.84 | 63.81 |
| blue dye | 8.88 | 8.49 |
| 99mTc+blue | **70.62** | 19.9 |
| blue + ICG +99mTc | 34.54 | **78.58** |
| 99mTC | 49.92 | 28.82 |

The place where the SUCRA value is bold denotes the value top 3 indicators.
